# A mechanistic pharmacokinetic model for intrathecal administration of antisense oligonucleotides

**DOI:** 10.3389/fphys.2023.1130925

**Published:** 2023-06-02

**Authors:** Andreas A. Linninger, Dipak Barua, Yaming Hang, Sergio Iadevaia, Majid Vakilynejad

**Affiliations:** ^1^ University of Illinois at Chicago, Chicago, IL, United States; ^2^ Takeda Pharmaceuticals, Cambridge, MA, United States

**Keywords:** intrathecal delivery, pharmacokinetic modeling and simulation, antisense oligodeonucleotides, central nervous system, computational fluid dynamics

## Abstract

Intrathecal administration is an important mode for delivering biological agents targeting central nervous system (CNS) diseases. However, current clinical practices lack a sound theorical basis for a quantitative understanding of the variables and conditions that govern the delivery efficiency and specific tissue targeting especially in the brain. This work presents a distributed mechanistic pharmacokinetic model (DMPK) for predictive analysis of intrathecal drug delivery to CNS. The proposed DMPK model captures the spatiotemporal dispersion of antisense oligonucleotides (ASO) along the neuraxis over clinically relevant time scales of days and weeks as a function of infusion, physiological and molecular properties. We demonstrate its prediction capability using biodistribution data of antisense oligonucleotide (ASO) administration in non-human primates. The results are in close agreement with the observed ASO pharmacokinetics in all key compartments of the central nervous system. The model enables determination of optimal injection parameters such as intrathecal infusion volume and duration for maximum ASO delivery to the brain. Our quantitative model-guided analysis is suitable for identifying optimal parameter settings to target specific brain regions with therapeutic drugs such as ASOs.

## Introduction

Intrathecal (IT) administration is a preferred delivery mode for small-molecule and biologics targeting CNS diseases including neuromuscular disorders (Bottros and Christo, 2014). IT administration is carried out by injecting a drug solution into the cerebrospinal fluid-filled subarachnoid space (SAS). This delivery mode has the crucial advantage over systemic delivery that it bypasses the blood-brain-barrier (BBB) so that active agents reach the brain via cerebrospinal fluid (CSF) route (Calias et al., 2014). Therefore, intrathecal delivery has emerged as a preferred modality for therapeutic proteins and biomolecule-based agents that cannot cross the BBB (Calias et al., 2014; Liddelow, 2018). Examples include antisense oligonucleotides (ASOs) that hold great promise for future treatment options of various CNS diseases, including Parkinson’s Disease, Spinal Muscular Atrophy, Huntington Disease, and Alzheimer Disease (Bennett et al., 2019; Scoles et al., 2019; Kuijper et al., 2021).

Unfortunately, severe knowledge gaps concerning achievable patterns of temporal and spatial biodistribution of IT administered drugs, especially for therapeutic proteins and biological macromolecules, cause delays in tapping the full potential of IT delivery in a clinical setting. This study was motivated by the need for a computational platform that could serve as an *in silico* tool to advance the quantitative understanding of intrathecally-administered drug pharmacokinetics and biodistribution. We introduce a *distributed mechanistic pharmacokinetic model* (DMPK) to predict drug dispersion and partitioning along the neuraxis and into the brain. We demonstrate the model’s predictive capability by analysing pharmacokinetics and biodistribution for an ASO in non-human primates and computing the effect of specific variables on the efficacy of delivery of an IT administration.

Current IT treatment practices lack a mechanistic foundation for correlating variables that determine drug- and subject-specific physiological properties to the efficacy of delivery (Hettiarachchi et al., 2011). Some of these variables include adjustable design conditions for an IT protocol, such as infusion time (duration of a bolus), infusion volume (bulk volume and drug concentration of the injected drug solution), frequency of administration and drug solution concentration (Penn and Kroin, 1987; Chen et al., 1999; Hettiarachchi et al., 2011; Xu et al., 2011; Belov et al., 2021). For example, duration and infusion volume generate convection-enhanced fluid flow in the SAS which in turn enhances biodistribution (BD) in the CNS. Moreover, proper drug dosage and scheduling frequency may be crucial to achieve therapeutically effective concentration at the target site of action (Kerr et al., 2001). Therefore, quantitative understanding of design parameters is imperative for their proper management aiming at intended and reproducible clinical outcomes. Unfortunately, current clinical practices rely on limited evidence and perspectives of individual clinicians (Hassenbusch and Portenoy, 2000) without the benefit of a quantitative theory for selecting these variables systematically.

There are several obstacles to purely experimental (*in vivo*) determination of optimal IT parameters values in a clinical setting. One challenge is to generalize outcomes from a limited number of trials and deconvolute the effects of multiple factors associated with the drug, patient, or procedural differences. Each drug agent has unique physicochemical properties such as molecular size and lipophilicity that determine biodispersion in the spinal fluid and absorption propensities into plasma or tissues (Bernards and Hill, 1992; Ummenhofer et al., 2000; Bernards et al., 2003). Subject-specific anatomical features as well as postural or positional differences of an IT administration introduce considerable experimental variability (Kerr et al., 2001; Coenen et al., 2019). There are also technical barriers to conducting repeated experiments over extended periods which necessitate high-precision control devices operating accurately and safely in living subjects. The cost and invasive nature of these experiments put further restrictions.

To complement clinical trial and error studies, mathematical models aim at predicting time dependent drug concentration trajectories after IT injection as a function of infusion parameters. In classical pharmacokinetics (PK) models, this task involves fitting of kinetic exchange parameters, typically black-box exponential dynamics (e.g., mono-exponential, bi-exponential curves), to dose-response data. Classical PK simulations (Ummenhofer et al., 2000; Eisenach et al., 2003) can reproduce dose-response curves for specific administration protocols, but extrapolate poorly when the dosing regime is changed, subject-specific differences need to be accounted for or when applied to a different cohorts of human subjects (male, female, child) or animal species.

Recently, a semi-mechanistic and an empirical model have been developed to analyse PK and BD characteristics of intrathecally administered ASOs (Biliouris et al., 2018; Monine et al., 2021). These two approaches were structurally similar although the second model offered more granularity by dividing CSF into three spatially distinct regions and including liver and kidneys as the peripheral organs. Both approaches demonstrated excellent agreement with observed PK and BD in non-human primates (NHP). However, none of the models can predict the infusion-induced spatiotemporal distribution of ASO along the neuroaxis. Moreover, these models cannot connect infusion variables to CSF flow to assess the resulting impact on drug pharmacokinetics and biodistribution. The shortcoming of classical pharmacokinetic-pharmacodynamic (PKPD) models is rooted in the fact that they do not explicitly incorporate mechanistic transport phenomena along the neuraxis (i.e., spatially distributed mechanistic models of drug transport). The effect of flow induced micro-mixing that leads to accelerate convective drug transport in the spinal compartment due the pulsatile flow and periods small periodic deformation along the neuraxis is missing.

As an alternative to purely classical (black box) PK modeling, computational fluid dynamics (CFD) methods can directly incorporate the effect of CSF hydrodynamics on drug dispersion in the CNS. Earlier CFD models aided in the developing a fairly accurate quantitative understanding of natural CSF flow characteristics in the spinal or cranial space (Loth et al., 2001; Kurtcuoglu et al., 2005; Gupta et al., 2008; Roldan et al., 2009; Gupta et al., 2010; Linge et al., 2010; Sweetman and Linninger, 2011; Rutkowska et al., 2012). Several models have been used to investigate IT infusion-enhanced transport and dispersion of molecules in the SAS (Tangen et al., 2015a; Tangen et al., 2017a; Tangen et al., 2020). Our group employed direct numerical simulation to predict drug dispersion after intrathecal injection (Tangen et al., 2015b; Tangen et al., 2016a; Linninger et al., 2016). Standard CFD methods were extended to enable the quantification of subject-specific CSF dynamics in the cranial and spinal subarachnoid space because *in-vitro* experiments demonstrated that amplitude and frequency of CSF pulsations were critical parameters for the observed rapid biodispersion of IT delivered tracers (Herriarachchi et al., 2011; Hsu et al., 2012). Moreover, microanatomical features of the spinal subarachnoid space were implicated in generating *geometry-induced* dispersion, a flow phenomenon active in the pulsatile spinal CSF that can explain why active agents move rapidly from the injection site towards the brain, despite the slow molecular diffusivity of most injection molecules and low Reynolds number flow regime in the spinal CSF.

Tangen et al. (Tangen et al., 2015a; Tangen et al., 2017b) used direct numerical simulation (DNS-CFD) to investigate the roles of SAS microanatomical features on the mixing and dispersion of intrathecally infused drug species along the neuroaxis and to account for chemical kinetics and uptake into the tissue. In separate works, it was demonstrated that larger injection volumes could more efficiently disperse radiolabelled tracer molecules into the rostral direction (Tangen et al., 2017a; Tangen et al., 2020). Unfortunately, the oscillatory CSF transport dynamics used in DNS-CFD models engender high computational cost/simulation time, which is suitable mainly for detailed studies on short time scales ranging few minutes to hours after the IT infusion. Time scale separation in a typical preclinical or clinical study could be a few minutes to several months between the dosing and data collection. Thus CFD models, which capture the microscale transport phenomena in subsections of the CNS, are often computationally too expensive to perform PK and BD predictions over a clinically-relevant time scales of days or weeks.

In order to predict BD of ASO over long periods at low computational cost, we propose here a reduced order model. The distributed mechanistic pharmacokinetic (DM-PKPD) model centers on an effective dispersion model along the neuroaxis (1D diffusive transport) to account for the effect of geometry induced mixing responsible for rapid caudocranial drug transport as well as axial bulk convection due to high injection volumes (1D convective transport). In addition, drug uptake and tissue interactions are incorporated using chemical kinetic approaches. Because main mechanistic principles of transport and reaction kinetics along the neuraxis are preserved, it is easy to scale and is flexible for the incorporation of key subject specific parameters such as CSF volume at very modest computation cost.

To demonstrate its practical value of DM-PKPD for the clinical trials, we incorporated ASO data used in a recent study (Monine et al., 2021). The fitted model captured spatiotemporal progression of ASO concentration in the CSF and spinal tissues and described long-term ASO profiles in the CNS and central body compartments with an acceptable accuracy. The model predicted impacts of volume and duration of an intrathecal administration on PK and BD over clinically relevant time scales. Our analyses indicate possible application of the model to optimize conditions for intrathecal administration and achieve target delivery to a site of action.

## Methods

Two subsections outline model structure and equations. Subsequent sections discuss simulation techniques and software used for model implementation followed by data used for calibration and analysis.

### Model structure

**FIGURE 1:**
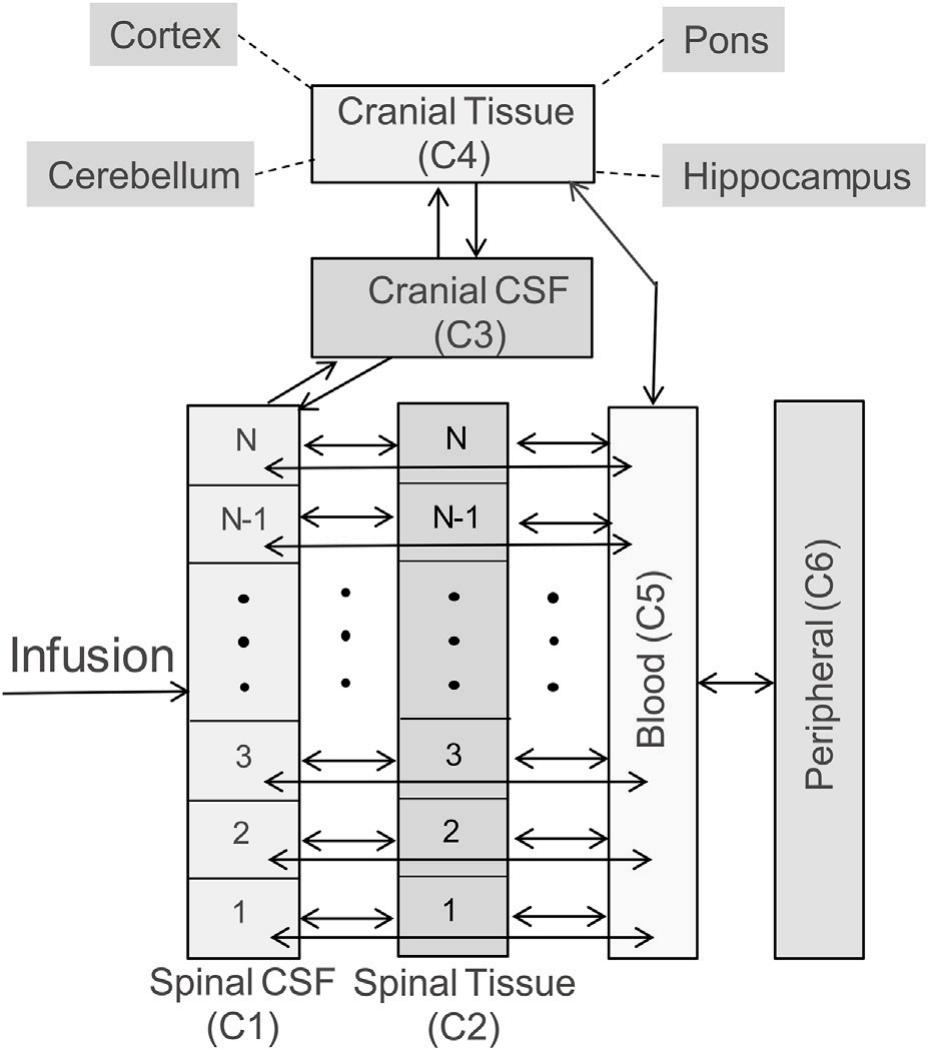
Schematic diagram illustrating the model structure. The model includes six compartments, representing CSF in the arachnoid space of the spine (labelled as *Spinal CSF, C1*), spinal cord (labelled as *Spinal Tissue, C2*), cranial CSF (labelled as *Cranial CSF, C3*), the brain (labelled as *Cranial Tissue, C4*), systemic circulation (labelled as *Blood, C5*), and peripheral tissues/organs (labelled as *Peripheral, C6*). The brain is further divided into four subcompartments: pons, hippocampus, cerebellum, and cortex. The double headed arrows in the diagram indicate reversible mass transfer connectivity among these compartments. The spinal CSF and spinal tissue compartments are spatially resolved, as indicated by *N* well-stirred volume elements, to implement the spatiotemporal model numerically, and account for spatial variation of concentration along the spinal axis. (Please see Methods for more details).

The whole-body model is structured to capture spinal transport of an IT-administered drug under advection and diffusion and its partitioning into the systemic circulation, the central nervous system (CNS), and peripheral tissue compartments as illustrated in the schematic diagram in Figure 1. The DM-PKPD model covers six anatomical regions: *Spinal CSF*, *Spinal Tissue*, *Cranial CSF*, *Cranial Tissue*, *Blood*, and *Peripheral* (Figure 1). *Spinal CSF* (denoted as compartment C1 henceforth) represents the spinal fluid-filled SAS spanning the entire spine. We conceptualize the CSF-filled spinal SAS as a cylindrical one-dimensional flow channel of a uniform cross-section. *Spinal Tissue* (C2) represents the spinal cord as a cylindrical rod with uniform cross section. The dimensions of these anatomical spaces are parameterized based on the physiological attributes of a typical adult non-human primate (NHP). We considered four more well-stirred compartments (lumped, no spatial distribution): *Cranial CSF* (C3) embodies CSF occupying the cranial space. *Cranial Tissue* (C4) represents the brain parenchyma, which was further divided into four sub-compartments, namely, *Pons*, *Hippocampus*, *Cerebellum*, and *Cortex*, to account for the heterogeneity among these regions. The *Blood* (C5) compartment stands for the systemic circulation. Compartment (C6) encompasses *peripheral* organs and body tissues, which are capable of absorbing the drug species from systemic circulation.

The bidirectional arrows in Figure 1 indicate reversible mass transfer fluxes among the compartments. The distributed *Spinal CSF* and *Spinal Tissue* compartments are spatially discretized along the axis using cylindrical finite volumes. The number of sub-volumes can be adjusted freely to obtain different degrees of spatial discretization (typically we used 50 to 100 sub-elements for discretizing the spinal CSF space). *Spinal CSF* elements are in contact with a corresponding adjacent *Spinal Tissue* element (Figure 1). All spinal CSF and tissue regions have access to the systemic *Blood compartment* (systemic circulation). The *Cranial Tissue*, *Blood*, and *Peripheral* compartments are also interconnected to enable possible drug transfer between them (Figure 1).

### Model equations

***a. Spinal CSF*** (***C1***) ***:*** *Spinal CSF* was modelled as a cylindrical flow channel with uniform crossection. The bottom (sacral) end of this fluid channel is closed, and the top (cervical) end is open to the cranial compartment. The channel is spatially discretized to numerically implement the governing partial differential equation (PDE) below:

$$\begin{array}{c} \frac{\partial C_{1}}{\partial t}=-\nabla \left(uC_{1}\right)+D\nabla^{2}C_{1}-k_{1}C_{1}-{\dot{m}}_{1,2}-{\dot{m}}_{1,5} \end{array}$$ (1)

Here, $C_{1}\left(x,t\right)$ is the local concentration at position x , which represents the distance from the sacral end. $u\left(x,t\right)$ is the local velocity at x due to the infusion-induced bulk flow in the channel. *D* is the effective diffusivity of the drug molecule in CSF and $k_{1}$ is the rate constant for first-order elimination by metabolism or non-specific tissue uptake. ${\dot{m}}_{1,2}\left(x,t\right)$ is the rate of absorption in C2 (*Spinal Tissue*). The term ${\dot{m}}_{1,5}\left(x,t\right)$ is the rate of ASO leakage into *Blood plasma* (C5). Mass transfer fluxes are determined by the local concentration difference between spinal CSF and the respective adjacent compartments. For example, the mass transfer ${\dot{m}}_{1,2}\left(x,t\right)$ is a function of mass transfer coefficients, U_12_, surface area of the interface, A_12_, and concentration difference, C_1_(t)-C_2_(t). Property values can be found in Supplementary Table S1. Spinal CSF (C1) is also connected to cranial CSF (C3) via a flux boundary condition allowing for solute exchange from the cervical region into prepontine CSF spaces. Because this exchange occurs at the boundary only, it is not listed in the differential form of Eq. 1.

The injection-induced bulk fluid velocity $u\left(x,t\right)$ was computed from the infusion rate and subject-specific dimensions of the cranial SAS. Axially uniform plug flow was assumed ignoring the radial velocity gradients arising from the oscillatory fluid-solid interactions in the deforming subarachnoid space (Tangen et al., 2020). The closed sacral end and fluid incompressibility assumption led to a unidirectional flow in rostral direction during infusion. The following equation was used to compute $u\left(x,t\right)$ resulting from an infusion with flowrate $\dot{F}\left(\bar{x},t\right)$ at location $\bar{x}.$

$$\begin{array}{c} u\left(x,t\right)=\frac{1}{A}\dot{F}\left(\bar{x},t\right)=\frac{1}{A}\int_{0}^{x}f\left(t,x^{\prime }\right)dx^{\prime } \end{array}$$ (2)

Here, A is the cross sectional area of flow channel, and $f\left(t,x\right)$ is the local infusion flux at position *x*. To avoid creating an unrealistic infusate source term at a single point (=unrealistic Dirac delta impulse), we define it as a distributed flux $f\left(t,x\right)$ by a spatial point spread function that symmetrically decays to zero within a small (2 cm) distance from the position of injection, $\bar{x}$ . This choice is based on the consideration that administration with a physical infusion catheter causes instantaneous distribution of the injected drug inside a small, but finite mixing zone around the point of injection. The local infusion function $f\left(t,x\right)$ can be related to the total infusion volume $V_{inj}$ based on the following equation,

$$V_{inj}=\int_{t1}^{t2}\int_{0}^{L}f\left(t,x^{\prime }\right)dx^{\prime }dt^{\prime }$$

where $t_{2}-t_{1}$ is the period of infusion. Eq. 2 describes a single infusion. For multiple injection doses, Eq. 2 is invoked each time for the duration of the additional infusion to compute the induced net bulk flow velocity $u\left(x,t\right)$ for the corresponding injection period.

The diffusion coefficient D in Eq. 1 represents the apparent effective biodispersion of ASO due to CSF pulsations. It accounts for a rapid dispersion of the injected agent due to natural pulsation of spinal CSF. Geometry induced mixing is a function of CSF pulsation amplitude and frequency and is orders of magnitudes larger than molecular diffusion as noted in our earlier work (Hsu et al., 2012; Hsu and Linninger, 2013; Tangen et al., 2015a; Tangen et al., 2016b; Tangen et al., 2017a) A first-order clearance rate is included to consider any unaccounted losses in spinal CSF. Mass transfer is allowed between *Spinal CSF* and *Spinal Tissue* driven by local concentration difference:

$${\dot{m}}_{1,2}\left(x,t\right)=U_{1,2}\;\left[C_{1}\left(x,t\right)-\beta_{1,2}C_{2}\left(x,t\right)\right]$$

An additional parameter 0≤β≤1 introduces a “trapping” or “sticky” effect in the spinal tissue to allow for a slow tissue release consistent with observation. Similar expression was used to allow reversible mass transfer between *Spinal CSF* and *Blood plasma*:

$${\dot{m}}_{1,5}\left(x,t\right)=U_{1,5}\;\left[C_{1}\left(x,t\right)-\beta_{1,5}C_{5}\left(t\right)\right]$$

***b. Spinal Tissue*** (***C2***) ***:*** The *Spinal Tissue* compartment is modelled as a cylindrical domain of the same length as C1 (*Spinal CSF*). Since molecular transport in the interstitial space is relatively slow, tissue diffusion along the neuraxis (=our x coordinate) has been omitted. The local concentration $C_{2}\left(x,t\right)$ at a specific position x in the spinal tissue compartment is described in (Eq. 3):

$$\begin{array}{c} \frac{dC_{2}}{dt}=-k_{2}C_{2}+{\dot{m}}_{1,2}-{\dot{m}}_{2,5} \end{array}$$ (3)

Parameter $k_{2}$ represents a first-order clearance in tissue. The other two terms represent mass transfer with *Spinal CSF* and *Blood* compartment respectively, as detailed in the previous section. The rod-shaped *Spinal Tissue* domain was uniformly discretized in the axial direction with the same number of elements as *Spinal CSF*. Each discrete volume element was connected to the adjacent volume element of *Spinal CSF* to allow for mass transfer driven by the local concentration difference between the two elements (Figure 1). Moreover, each volume element *Spinal Tissue* was also linked to the *Blood* compartment (systemic circulation) to allow for solute exchange based on the difference in concentration between the two (Figure 1). This choice was supported by the observation of rapid appearance of ASO into blood plasma after IT administration.

***c. Cranial CSF*** (***C3***) ***:*** We model *Cranial CSF* as a well-mixed lumped compartment, where concentration is defined by the following ODE:

$$\begin{array}{c} \frac{dC_{3}}{dt}=-k_{3}C_{3}+{\dot{m}}_{1,3}-{\dot{m}}_{3,4} \end{array}$$ (4)

Parameter $k_{3}$ is a first-order clearance coefficient. The expression

$${\dot{m}}_{1,3}=U_{1,3}\left(C_{1}\left(L,t\right)-C_{3}\right)$$

accounts for mass transfer with the top (cervical) edge of C1 (*Spinal CSF*). The second flux

$${\dot{m}}_{3,4}=U_{3,4}\left(C_{3}-\beta_{3,4}C_{4}\right)$$

represents the rate of mass transfer with *Cranial Tissue* (brain).

***d. Cranial Tissue*** (***C4***) ***:*** We model the aggregate brain or cranial tissue as a single compartment and describe its concentration using the equation below:

$$\begin{array}{c} \frac{dC_{4}}{dt}=-k_{4}C_{4}+{\dot{m}}_{3,4}-{\dot{m}}_{4,5} \end{array}$$ (5)

Parameter $k_{4}$ represents a first-order clearance. Moreover, it is allowed to exchange mass with *Cranial CSF* ( ${\dot{m}}_{3,4})$ , as discussed above. Additionally, it is also directly connected to *Blood* and allowed to exchange mass at a rate

$${\dot{m}}_{4,5}=U_{4,5}\left(\beta_{4,5}C_{4}-C_{5}\right)$$

***e. Blood*** (***C5***) ***:*** This compartment represents the plasma or systemic circulation and it is described by the following ODE:

$$\begin{array}{c} \frac{dC_{5}}{dt}=-k_{5}C_{5}+{\dot{m}}_{1,5}+{\dot{m}}_{2,5}+{\dot{m}}_{4,5}-{\dot{m}}_{5,6} \end{array}$$ (6)

The first term on the right-hand side accounts for a first-order clearance. The next three terms account for mass transfer with *Spinal CSF*, *Spinal Tissue*, and *Cranial Tissue*, respectively, as described in the previous sections. The final term represents solute exchange with the peripheral compartment:

$${\dot{m}}_{5,6}=U_{5,6}\left(C_{5}-\beta_{5,6}C_{6}\right)$$

***f. Peripheral*** (***C6***) ***:*** The peripheral compartment is described by the following ODE:

$$\begin{array}{c} \frac{dC_{6}}{dt}=-k_{6}C_{6}+{\dot{m}}_{5,6} \end{array}$$ (7)

The two terms on the right-hand side describe a first-order clearance and mass transfer to the plasma (*Blood*) compartment, respectively.

***g. Brain and functional cerebral sub-domains:*** The *Cranial Tissue* compartment is divided into four sub-domains: *Pons*, *Hippocampus*, *Cerebellu*m, and *Cortex*. This division enables consideration of regional heterogeneity in PK and BD events in different brain regions. The drug in *Cranial Tissue* is distributed among these four regions based on a simple volumetric partition rule:

$$\begin{array}{c} C_{i}\left(t\right)={\frac{1}{V_{i}}\phi_{i}V}_{4}C_{4}\left(t\right) \end{array}$$ (8)

Here, $C_{i}$ and $V_{i}$ respectively stand for the concentration and volume of each sub-domain. $C_{4}$ and $V_{4}$ represent the concentration and volume of *Cranial Tissue* (C4). The parameter $0\leq \phi_{i}\leq 1$ is the partition coefficient associated with sub-domain *i*. Note that $V_{4}=\underset{i}{\sum }V_{i}.$ and $\underset{i}{\sum }\phi_{i}=1$ . The volumes of *Pons*, *Hippocampus*, and *Cerebellum* have been reported and the volume of *Cortex*, which is a generic representation of the rest of the brain in our model, is obtained by subtracting these three volumes from $V_{5}$ . The unknown partition coefficients were identified by fitting the model to corresponding PK data (please see the Results section for more details).

### Simulation and software

The original software developed by the corresponding author (AL) for this project was adapted for this study. A finite volume scheme was used to discretise axial transport along the neuraxis to convert the spatially distributed PDE system in Eq. 1 into a set of ODEs. The *Spinal CSF* and *Spinal Tissue* compartment were uniformly discretized into *N* volume elements (*N* = 100 used for all simulations). These 2*N* discrete volumes and four well-stirred compartments led to a system of (2*N* + 4) ODEs. The model was scripted in MATLAB (Version 2020a). Time integrations were performed using MATLAB-built in solver ode45.

### Data sources and calibration

In the mechanistic model, the majority of parameters have anatomical or biochemical meaning so that values can be set to physiological ranges. For example, volumes, cross sectional areas, surface exchange, etc. were taken from medical images and prior studies listed Supplementary Table S1. Mass transfer coefficients and stickiness parameter (β′s) were adjusted by repeated simulations. Simulation-based trial-and-error calibration gave excellent fit to NHP ASO PK data in spinal CSF, plasma, and different brain compartments for all dosing regimes with final parameter choices listed in Supplementary Table S1.

Kinetic uptake rates, k_i_, were calibrated using PK data for class of 2′-MOE and PS modified gapmer ASOs in cynomolgus monkeys reported in a recently-published study (Monine et al., 2021). Numerical values for these data were obtained by digitizing relevant plots in Monine et al. (Monine et al., 2021). Cerebral partition coefficients compartments were inferred from cerebral biodistribution data. Rigorous parameter estimation was therefore not necessary and deemed outside the scope of this study. For a detailed description of rigorous parameter estimation in distributed systems we refer to prior work by the corresponding author (Kulkarni et al., 2006; Zhang et al., 2007; Somayaji et al., 2008).

## Results

### ASO PK and biodistribution: Prediction vs data

**FIGURE 2:**
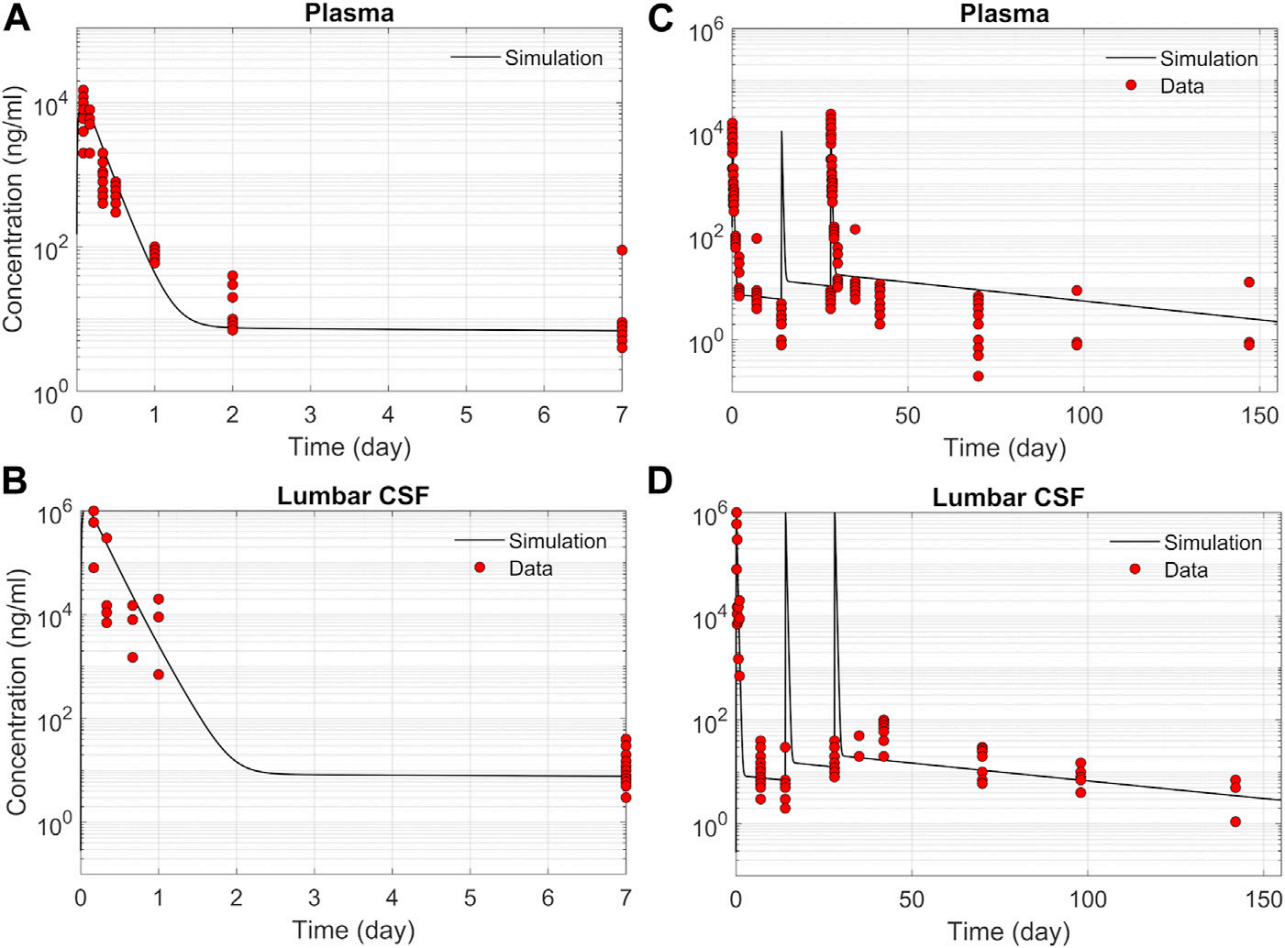
Comparison of the model fit with individual ASO PK data (Monine et al., 2021). **(A)** Plasma concentration-time profile over 7 days following IT administration of a single 12 mg dose. **(B)** CSF concentration-time profile in the lumber region over 7-day following IT administration of a single 12 mg dose. **(C)** Plasma concentration-time profile following IT administration of three repeated 12 mg doses applied at 2-week intervals. **(D)** CSF concentration-time profile in the lumbar region following IT administration of three repeated 12 mg doses applied at 2-week intervals.

**FIGURE 3:**
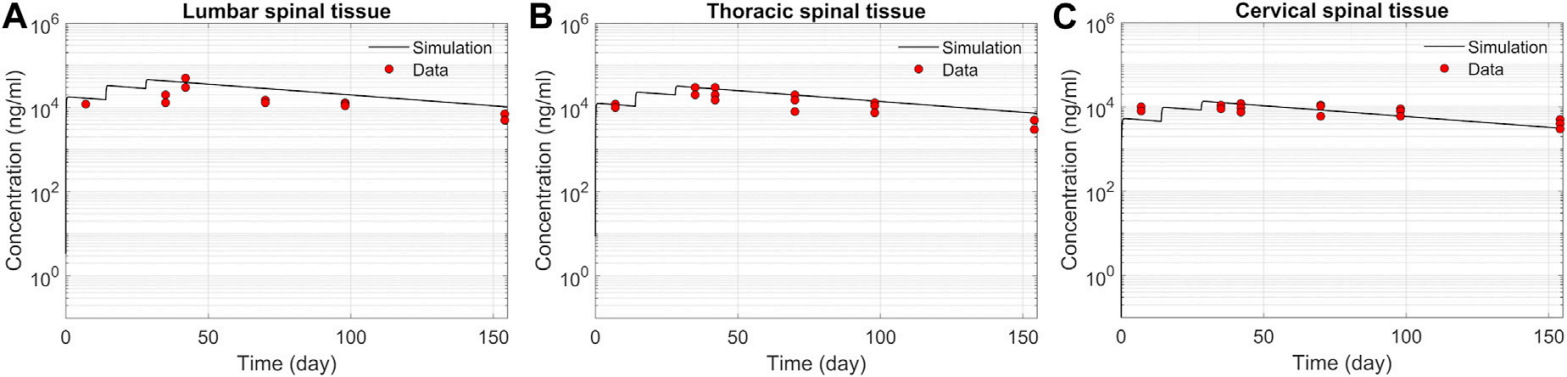
Comparison of the model fit for spinal tissue PK data. The points represent data (Monine et al., 2021), and the lines represent simulations. Responses after three doses are shown for **(A)** lumbar, **(B)** thoracic, and **(C)** cervical spine. The PK data correspond to three repeated 12 mg doses applied at 2-week intervals with the first dose beginning at time zero.

**FIGURE 4:**
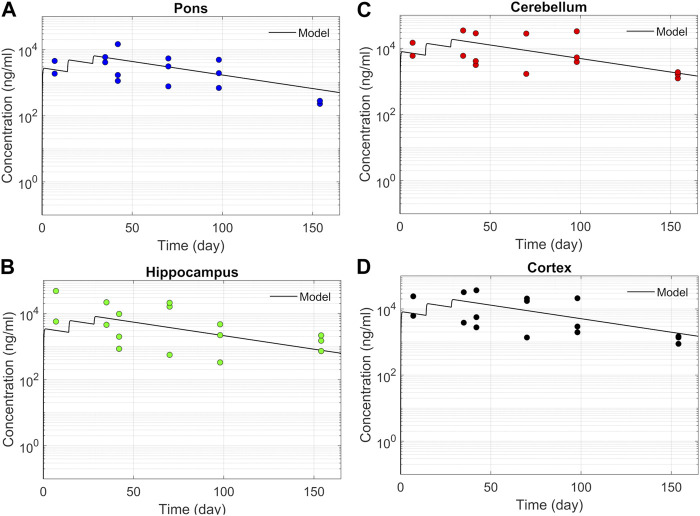
Model fit comparisons for PK in the brain tissues. Points indicate data and lines indicate simulations. Comparisons are shown for four brain regions **(A)** Pons, **(B)** Hippocampus, **(C)** Cerebellum, and **(D)** cortex. The data correspond to PK against three 12 mg repeated doses applied at 2-week intervals.

We tested the proposed DMPK model to characterize ASO dispersion and partitioning along the neuraxis. We calibrated mass transfer, and pharmacokinetic parameters to test its predictive performance against the ASO PK reported in these previous studies (Monine et al., 2021). Figure 2 shows the model predictions fitted to the observed ASO PK in plasma and CSF in the lumbar spine. These results represent responses against a single dose of 12 mg ASO administered in the lumbar region (Monine et al., 2021). The volume of the infused drug solution was 1 mL administered over 1 min. Figures 2C,D show long term responses for a duration of a week up to 150 days against three repeat doses of the same configuration. The doses were repeated at 2-week intervals as indicated. The plot shows that the predictions were in good agreement with all datasets. Some deviation was noted at the lower concentration regimes (<10 nm). Such departures were also observed in prior models (Monine et al., 2021). Figure 3 compares the model’s best fit to the reported data for ASO PK in spinal tissue at the lumbar, thoracic and cervical region (Monine et al., 2021) after three injection doses administered in 2 week intervals over a period of more than 150 days Figure 4 compares for the ASO concentrations accumulated in the four different brain regions: pons, cerebellum, hippocampus, and cortex. The results recorded in these figures show that DMPK model tracked tissue PK profiles across various regions of the CNS reasonably well. The model calibrated model parameters capturing the observations led the parameters listed in Supplementary Table S1. We also inferred the effective ASO diffusivity in CSF, *D* ≈ 0.1 cm^2^/min. This value is orders of magnitude larger than typical molecular diffusion one would expect based on the molecular weight of ASO and water-like thermophysical properties of CSF. As noted in earlier studies, *D* is not the *molecular diffusion coefficient,* but rather represents an apparent dispersion coefficient arising from the geometry induced mixing effects due to natural CSF pulsations. It should be noted that the value inferred for *in vivo* monkey data (NHP) here is much smaller than an independent, experimentally determined dispersion rates of tracers determined for human conditions (*in vitro* human effective dispersion coefficient *D* ≈ 1.53–4.7 cm^2^/min^45^). Mass transfer coefficients associated with mass exchange between different compartments were also derived from calibrations as listed in Supplementary Table S1. The stickiness parameters, β , which quantify the trapping capacity of the tissue compartments, were unknown in the model and identified from calibration (Supplementary Table S1). The four partition coefficients, each associated with a distinct brain region, were also identified by calibrating the model against data specific to these regions (Figure 4).

### Spatiotemporal ASO dispersion in the spine

**FIGURE 5:**
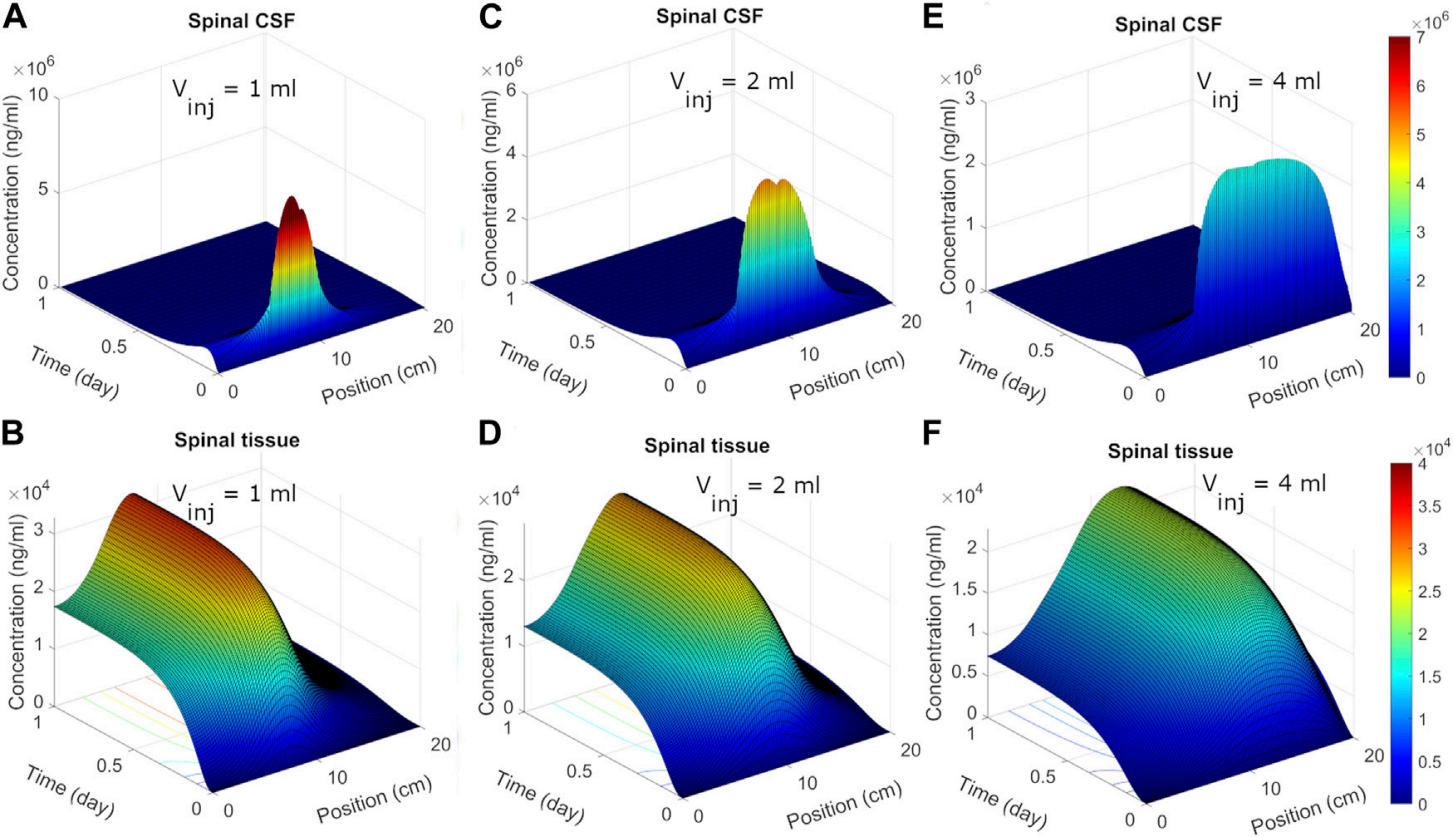
Predicted post-infusion spatiotemporal ASO distribution along the neuraxis in cyno monkey. The top and bottom panels show temporal evolution of concentrations in spinal CSF **(A, C, E)** and tissue **(B, D, F)**, respectively, in response to a single 1-mL, 2-mL, or 4-mL infusion. In each plot, position zero corresponds to the sacral end, and position at 20 cm indicates the cervical end. Simulations were performed considering 1-min of bolus administration in the lumbar part of the spine. A fixed dose (12 mg) of ASO was assumed regardless of the infusion volume.

We next used the calibrated model to predict spatiotemporal distribution of spinal ASO uptake under different infusion scenarios. The surface plots in Figure 5 show predicted ASO concentration as a function of time and spinal position in response to a single IT administration of 12 mg ASO. In the simulations, the dose was followed by a brief flush with ASO-free media per the protocol described by Monine (Monine et al., 2021). The ability to predict the impact of different infusion modes on the spatiotemporal pattern of ASO concentration profiles along the neuroaxis is a major advantage of the DMPK approach over the prior models. Predictions are shown under three different infusion scenarios each involving a distinct infusion volume (1, 2, and 4 mL, respectively). Other than the infusion volume, all conditions were identical. The infusion dose and duration were fixed at 12 mg and 1 min, respectively. The flush involved a 1-min bolus of 0.25 mL ASO-free solution (Monine et al., 2021).

Under 1 mL infusion, the model predicted a narrow distribution of ASO in CSF near the infusion site (Figure 5A). Initially, the drug disperses fast under the influence of the forced injection. During high volume injection which generated considerable convective drive, the predicted distribution profile gradually advanced into the cranial direction as long as an infusion (drug solution or flush) persisted. After cessation of the infusion, peak distribution became stagnant, because the induced bulk velocity vanishes. The concentration profile continued to spread under effective diffusive forces due to CSF pulsations, thus allowing ASO dispersion to continue both in the caudal and rostral directions. In the present simulation run, ASO concentrations in CSF decayed before substantial amount could reach the cervical and cranial region. At the same time, predicted ASO uptake to the spinal tissue was substantial (Figure 5B). This wide dispersion of ASO into the spinal tissue reflects its continued uptake from the CSF to spinal tissue. As ASO molecules in CSF continued shifting under convective forces, greater areas of the spinal tissue got exposed. This led to long-lasting absorption into spinal tissue with wider distances travelled by ASO in CSF before the infusions ceased. With higher infusion volume of infusion (Figures 5C–F), an even large portion of the neuroaxis could be achieved. The convective forces under 2- or 4-mL infusion allowed drug molecules to travel further into cervical direction thus exposing larger areas (∼75% and 100%, respectively) of the spinal tissue to significant ASO doses (Figure 5).

### ASO delivery to the brain

**FIGURE 6:**
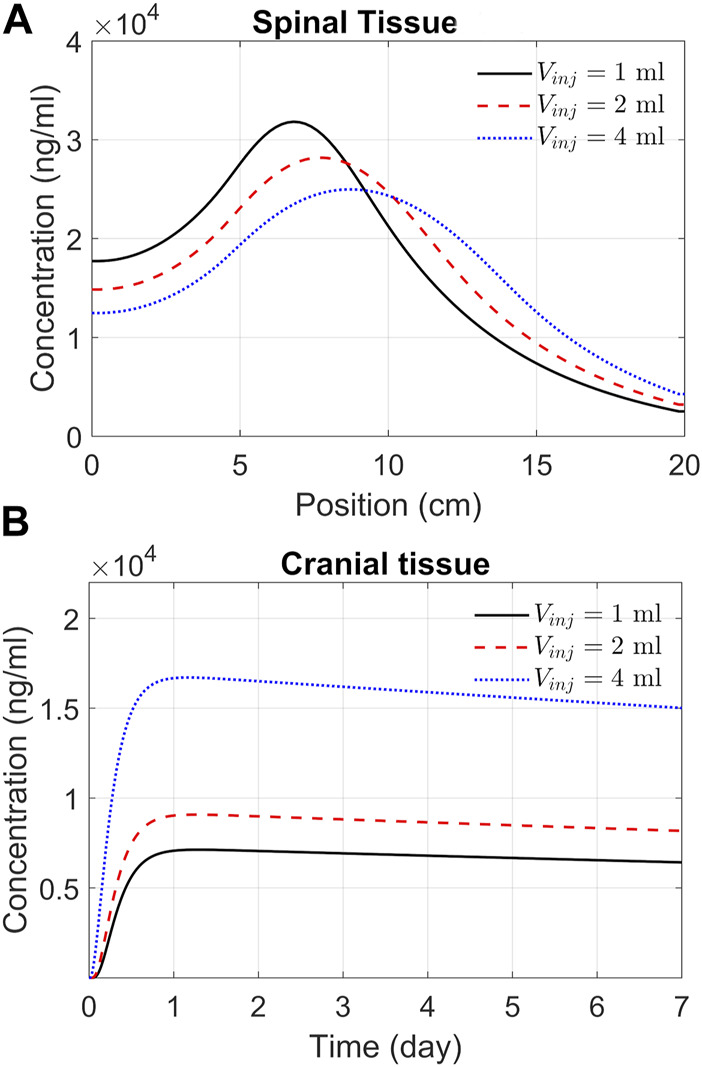
Predicted effects of infusion volume on tissue PK. **(A)** ASO concentration in spinal tissue by the end of Day 7. Each curve corresponds to a different volume of infusion (1, 2, or 4 mL, as indicated). The X-axis represents position along the spine, where position zero and 20 cm correspond to the sacral and cervical end, respectively. **(B)** Concentrations in the cranial tissue as a function of time under the above three infusion volumes. Simulations were carried out considering a 1-min bolus administration of 12 mg ASO regardless of the infusion volume.

The previous section addressed spatiotemporal ASO dispersion in the spinal tissue under three different infusion volumes. In drug therapies targeting the brain, spinal tissue uptake represents a loss. To get a better understanding of spinal and cranial targeting ability, we investigated ASO concentration profiles along the neuroaxis. The results of Figure 6A demonstrate that higher infusion volume causes wider dispersion along the neuroaxis and induce a shift of peak concentrations and gradients in cranial direction (shown in the previous section). The cranial advancement of these gradients is necessary to achieve a considerable increase in the cranial delivery at higher infusion volumes, as depicted in the plots in Figure 6B.

### Effect of the infusion rate and duration on spinal and cranial delivery

We next used the DMPK model to investigate the impact of infusion rate and duration on the efficacy of delivery. This addressed how an infusion of the same volume of ASO solution at different rates affects ASO delivery to the brain. For instance, we may consider infusing 12 mg of ASO in a 1 mL solution at a rate of 1 mL/min. Alternatively, the same dose could be applied considering infusion at a rate of 0.01 mL/min. High volume injection generates larger convective forces, yet the higher injection impulse is sustained only for a minute. In contrast, slow infusion has low injection impulse for a longer duration of 100 min. The goal was quantifying the trade-off between infusion rate and duration.

**FIGURE 7:**
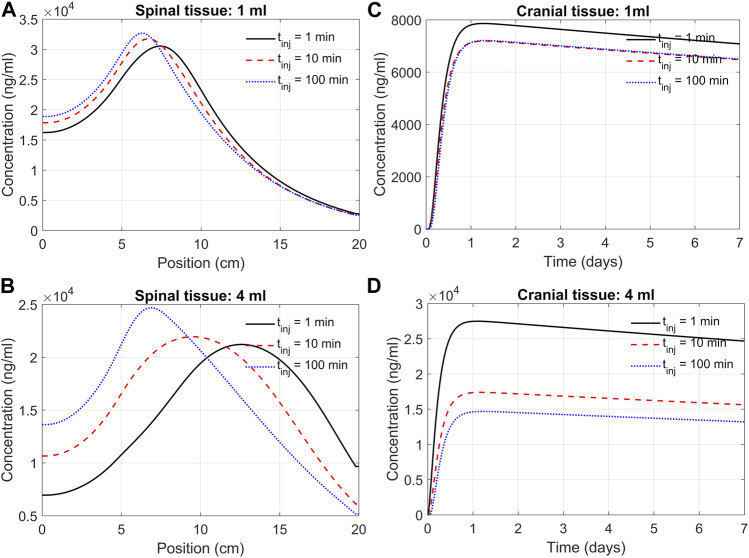
Predicted effects of infusion duration on tissue PK observed at day seven after administration. **(A)** Concentration in the spinal cord at day 7. Each curve corresponds to a different infusion time (1-min, 10-min, or 100-min, as indicated) but the same infusion volume of 1 mL. The X-axis indicates spinal position with position zero being the sacral end and position at 20 cm being the cervical end, respectively. **(B)** Profiles as described in Panel A but for a infusion volume of 4 mL. **(C)** Cranial tissue concentration under different infusion durations (1 min, 10 min, or 100 min, as indicated) but same infusion volume (1 mL). **(D)** The same as Panel C except for the infusion volume was 4 mL. Under all conditions, ASO amount was fixed at 12 mg regardless of the infusion time or volume chosen. All curves observed at day 7.

Figure 7 shows the model predictions under 1 min, 10 min, and 100 min infusion times. Our predictions support the notion that infusion rate (or infusion volume with appropriate drug dosing) has a substantial effect on cranial delivery. At a rate of 1 mL infusion, the duration of infusion had marginal impact on spinal dispersion or cranial delivery (Figures 7A,C). Under 4 mL infusion, a shorter infusion time shifted ASO distribution in the spinal tissue further into the cervical direction ((Figure 7B). However, with 4 mL infusion, high/fast infusion rates drastically improved cranial delivery (Figure 4D). These results indicate that the effect of infusion duration (or rate) cannot be decoupled from the infusion volume; unpredictable clinical outcomes may arise for the same dose when administered with variable duration and volume of an infusion.

### Optimal regimen for infusion volume and infusion time

**FIGURE 8:**
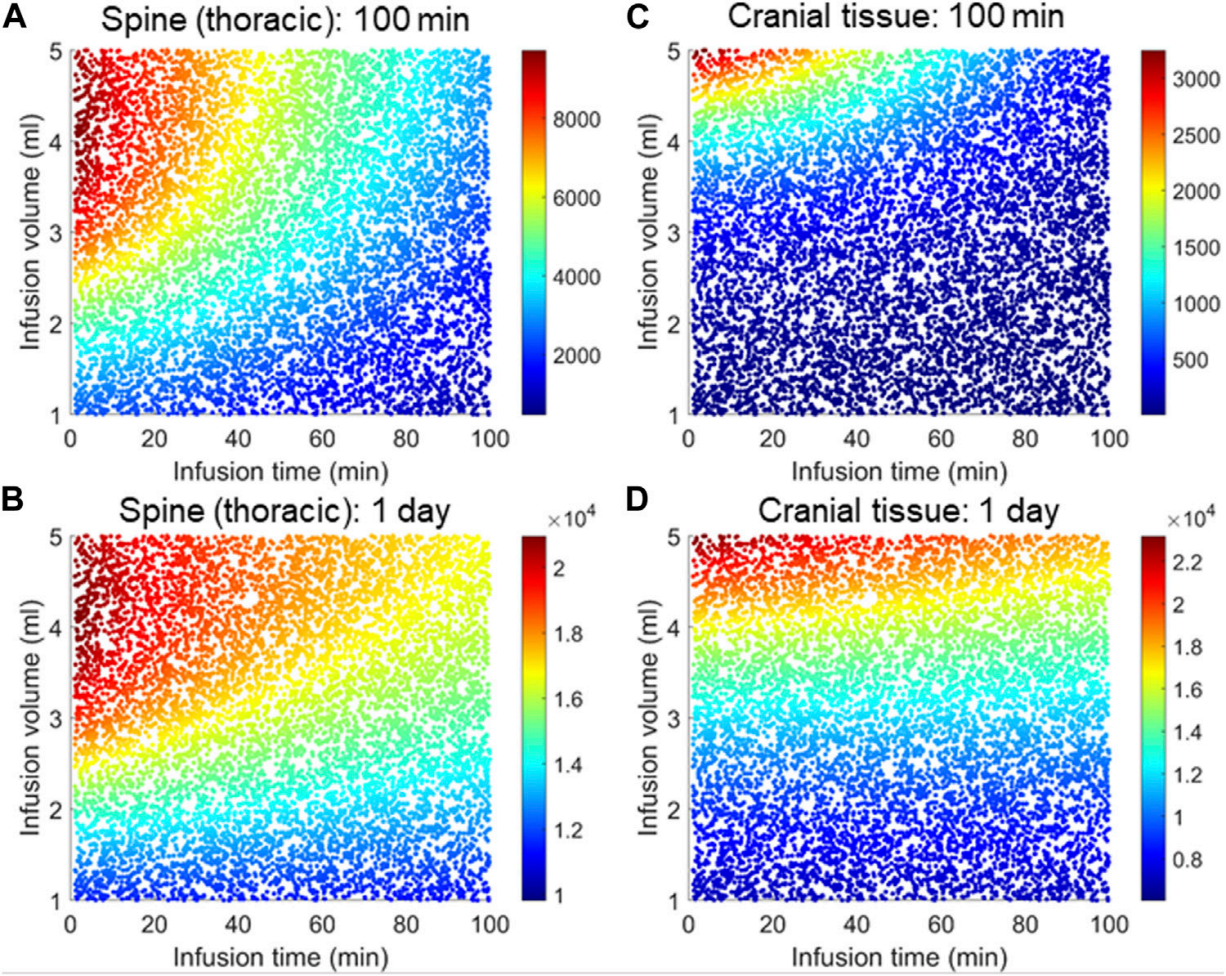
Combined effects of infusion volume and time on tissue distribution. Each panel corresponds 8,000 simulations each for a distinct combination of infusion time and infusion volume randomly sampled from the range specified by the X-and Y-axis, respectively. The color map indicates relative concentrations. **(A)** and **(B)** represent the thoracic region spinal tissue, **(C)** and **(D)** represent the cranial tissue at the indicated times. In all simulations, time zero is marked by the start of the infusion. A fixed amount (12 mg) ASO was considered regardless of the infusion volume or infusion time.

In this section we systematically investigate the combined effects of infusion volume and flow rate. We randomly sampled their values between 1 and 5 mL and 0.1–100 min, respectively, and performed simulations for each random combination. Each panel in Figure 8 represents 8,000 such simulations representing random combinations of these two variables.

The results in Figures 8A,B indicate that the effects from infusion time on the spinal tissue distribution could become apparent if infusion volume exceeds 1.5 mL. The cranial delivery was, however, less sensitive to infusion time (Figures 8C,D). The infusion volume should be at least 3 mL for the infusion time for ASO to reach the brain in therapeutic doses (Figure 8D).

Figure 8D provides insights into the optimal regime for these two input variables, which lies in the upper left corner of Figure 8D, where infusion volume is higher than 4.5 mL and infusion time shorter than 10 min. There are practical limitation for choosing this range. Infusion of a large volume within a short period could be invasive and avoided for safety reasons. Nevertheless, because cranial delivery appears much less sensitive to infusion time, it would still be beneficial to administer a higher volume over a longer time based on the upper-right corner of Figure 8D, or try accelerating drug dispersion by continuous injection or flushing of drug free artificial CSF to promote faster caudocranial transport.

## Discussion

This work introduces a distributed mechanistic pharmacokinetics (DMPK) model that can serve as a computational platform to simulate IT administration and enable predictive analysis of drug pharmacokinetics in the CNS.

The DMPK model was formulated keeping two primary objectives in mind: 1) capture the spatial distribution of transport phenomena along the elongated neuraxis; and 2) establish mechanistic convection-advection-reactive type transport equations that account for the effect of injection location, impulse and low rate in addition to geometry induced mixing effects with the potential inclusion of biochemical reaction kinetics. The key innovation over previous classical pharmacokinetic approaches is an explicit mechanistic description of the effects of pulsation-enhanced mixing on effective solute transport in the spinal CSF spaces. This enables the deployment of physiological parameters for transport phenome (convection and diffusion) instead of fitting “black-box” compartmental exchange coefficients. The mechanistic foundation enables the application of physics-based scaling laws to account for subject-specific variations or translation of data across different species (e.g., from monkey to humans) as well as the ability to reasonably captures effects of infusion modes, doses and scheduling on drug dispersion along the neuraxis. The convection term due to high volume injection and an effective dispersion relationship allowed us to quantify the relation of the injection mode (IT infusion), physiological CSF conditions (CSF pulsations and amplitude) on long-time ASO biodistribution in the CNS.

We used the model to determine parameters that quantify PK and biodistribution of an ASO-based therapeutic agent in non-human primates. The model-based analyses led us to identify partition coefficients dictating relative distributions of the ASO into four distinct regions of the brain parenchyma. Our results show that the model could serve as a predictive guide to optimize important variables that define the design conditions for an IT administration. In addition to capturing the complexity of infusion-induced spatiotemporal changes in the CSF and spinal tissues, the model demonstrated excellent agreement with the ASO pharmacokinetics and biodistribution reported in recent studies (Figures 2–4).

The mechanistic transport formulation is generic so that different drug compound administered intrathecally can be simulated. Due to the difference in the physiochemical properties, different drug molecules may display considerably distinct transport characteristics in the CSF and partitioning propensities into various compartments. Such differences could be more pronounced between small molecule compounds (e.g., many of the analgesics) and large biomolecule-based agents (such as large therapeutic proteins or ASOs). Smaller molecules might have faster dispersion rates in CSF and hence infusion-dictated flow might have weaker influence on its spinal dispersion. Differences in liphophility of drug molecules also alters their relative bioavailability and tissue uptake. It would be interesting to investigate to what extent parameters and partition coefficients identified in this study might be generalized to other ASOs or biomolecule-based agents having similar physiochemical properties.

Spinal tissue acts on administered drug like a retaining reservoir (=sink). Rapid infusion of ASO in spinal CSF leads to rapid increase in concentration (Figure 2). Some of the ASO is transferred to spinal tissue where ASO clearance is much slower. When ASO concentration in spinal CSF eventually decreases, ASO from spinal tissue re-enters in spinal CSF and can eventually advance to cranial CSF/tissue. Spinal tissue uptake is therefore able to delay caudocranial advance of solutes administered in the CSF. This effect is much stronger in smaller lipophilic drugs such as morphine than for large molecular weight ASO with slow rapid tissue uptake.

Our current analysis concerned PK and BD data from NHPs, and the model parameterization represents the nominal physiological attributes of an adult NHP subject (Supplementary Table S1). For our mechanistic model parameters, translation to human conditions is straightforward by simply adjusting anatomical differences and physiological attributes. The model could also accommodate individual patient variability of anatomical features. For example, between an adult human and a child, the spinal length could be significantly different, which may lead to considerable difference in the transport and dispersion behaviour in the spine under the same conditions of an IT administration. Alternative parameterization of the model is possible to capture such interindividual variability by creating virtual patient phenotypes.

Our predictions and analysis provide insights into the two important variables - infusion volume and duration - on the efficiency of cranial delivery. Predictions revealed that higher infusion volume can enhance drug dispersion along the spinal axis due to the higher convective forces from injection impulse and promote cranial delivery (Figures 7, 8). On the other hand, a shorter bolus may be more conducive to cranial delivery compared to a long or sustained infusion for the same infusion volume (Figures 6, 8). Nevertheless, the infusion volume and rate (or duration) should be chosen carefully considering the physiologically viable ranges associated with safety and tolerability. The positive impact of a higher infusion volume has also been reported in a recent study that investigated MALAT1 and MAPT ASO distribution in cynomolgus monkeys (Sullivan et al., 2020). Comparing under 1.8 mL and 0.4 mL IT bolus, this study found the higher infusion volume led to an increased accumulation of both ASOs in the rostral CNS (Sullivan et al., 2020). Consistent with our findings with a reduced order DMPK model here, a more rigorous DNS-CFD modelling study by Tangen et al. (Tangen et al., 2017a) reported that a higher infusion volume could augment the dispersion of tracer molecules along the spinal axis. The DNS-CFD had the advantage that it could predict the effective dispersion from first principles, while here the effective dispersion had to be inferred from experimental data. However, the DNS-CFD computations are much more CPU time consuming. Our DMPK model presented here is better suited for drug administration analysis over weeks or parametric sensitivity studies (=optimization of infusion settings and dosing as in the examples for Figure 8). An interesting combination would be to infer effective dispersion coefficients by rigorous DNS CFD or *in vitro* experiments (Ayansiji et al., 2022) and use the results with the DMPK-model for long term predictions and therapy optimization.

We further decided to investigate combined effect of infusion volume and duration to identify their ideal combinations aiming at higher delivery efficacy to the brain (Figure 8). Considering the physiological limitation for safe infusion, our predictions indicate the optimal range to be >3.5 mL for infusion volume and $0\leq t_{\inf}\leq 10$ min for infusion time for NHPs. These values are expected to be considerably different for human therapies. Studies suggest that both NHP and humans can tolerate IT bolus of 33%–42% of the total CSF volume (Rieselbach et al., 1962; Begley, 2004). This corresponds to maximum possible infusion volume of 3.5–4.5 mL for a NHP and 30–66 mL for a human considering typical CSF volume for corresponding species. On the other hand, typical intrathecal administration could take less than a minute to several minutes. However, a large bolus within a short time could be invasive and not recommended due to safety considerations (Belov et al., 2021).

Besides infusion volume and duration, other input variables might be the position of the injection site in the spine and frequency of repeat infusions. Our simulations suggest potential benefits than can be achieved by flush injections with artificial CSF (no drug) to accelerate caudocranial of previously administered drugs. This point was not investigated in detail in this study, although DMPK is able to quantify the effect of any infusion or dosing scenario on cranial delivery.

Our one-dimensional, hence less computationally demanding, approach overcame the computation time bottleneck of CFD which has limited utility in describing PK data at the clinically relevant time scales. DMPK excluded the microdetails of pulsatile fluid-solid interactions and short time scale fluid flow characteristics (Tangen et al., 2015a). Model reduction in the spatial dimension (=1D rather than 3D) domains did not compromise the model’s predictive ability to explain both short and long-term dispersion, PK and biodistribution in various compartments. This indicates that considering only effective dispersion in addition to bulk flow due to injection may be sufficient to adequately predict the impacts of IT infusion conditions on ASO PK along the neuroaxis and other body compartments.

### Limitations of the study

The spatiotemporal aspect of the model is implemented in a spatial and temporal coarse-grained approach (=1D flow field along the neuraxis with no oscillatory flow). Hence, the microscale solid-fluid interactions and transport phenomena inside the complex geometry of the spinal subarachnoid space is not captured. Instead, focus has been directed to bulk fluid flow and molecule dispersion so that computation could be manageable to perform simulations over clinically relevant time scales of weeks to months. The distributed portion (PDE transport model) was limited to the spinal axis only. Other regions, including the brain itself, are represented as lumped “well-mixed” compartments. This ignores the spatial non-uniformity of the brain parenchyma, which is heterogeneous in terms of accessibility of intrathecally administered molecules.

Compliance of the connected cranial and spinal CSF spaces, theoretical mass conservation and clinical studies suggest that the spinal cavity undergoes periodic expansion (Linninger et al., 2009; Sweetman and Linninger, 2011). Our original version of the DMPK model also has a deformable spinal compartment with adjustable spinal compliance (=fluid-structure interaction). However, the current study switched off deformations (=rigid cranial CSF compartment).

### Study highlights

Current clinical practices lack quantitative understanding of variables that govern the delivery efficacy of an IT administration. This work develops a fluid dynamic model to predictively investigate the pharmacokinetics, biodistribution, and delivery efficacy of an IT-administered drug compound. The model-based analysis provides quantitative information about the transport and pharmacokinetic parameters of a published ASO molecule. It predictively identifies the optimal combinations of an infusion volume and duration for this ASO molecule to maximize its delivery to the CNS. The platform may serve as a guide to determine drug- and subject-specific selection of optimal conditions for an IT protocol design.

## Data Availability

The original contributions presented in the study are included in the article/Supplementary Materials, further inquiries can be directed to the corresponding author.
