# Supplementary material for: A mechanistic pharmacokinetic model for intrathecal administration of antisense oligonucleotides
Source: Front Physiol. 2023 Jun 2;14:1130925. doi: 10.3389/fphys.2023.1130925 (PMC10272745; doi:10.3389/fphys.2023.1130925)
Supplement: Supplementary file 1 [file Table1.docx]

SUPPLEMENTARY MATERIALS

*Table S1: Model parameters (cyno monkey)*

| Parameter | Value | Unit | Comment |
| --- | --- | --- | --- |
| V_1_ | 8 | cm^3^ | ^a^Volume of spinal fluid (C1)[^46^](#_ENREF_46)^,^ [^50^](#_ENREF_50) |
| V_2_ | 8 | cm^3^ | ^b^Volume of spinal tissue (C2)[^21^](#_ENREF_21) |
| V_3_ | 3 | cm^3^ | ^c^Volume of cranial CSF (C3)[^46^](#_ENREF_46)^,^ [^50^](#_ENREF_50) |
| V_4_ | 65 | cm^3^ | ^d^Volume of cranial tissue (C4)[^21^](#_ENREF_21) |
| V_5_ | 180 | cm^3^ | ^e^Volume of blood/systemic circulation (C5) [^51^](#_ENREF_51) |
| V_6_ | 73 | cm^3^ | ^(This work)^Volume of peripheral compartment (C6) |
| V_pons_ | 1.5 | cm^3^ | Volume of subcompartment representing pons[^21^](#_ENREF_21) |
| V_hipp_ | 1.5 | cm^3^ | Volume of subcompartment representing hippocampus[^21^](#_ENREF_21) |
| V_cerb_ | 8 | cm^3^ | Volume of subcompartment representing cerebellum[^21^](#_ENREF_21) |
| V_cort_ | 54 | cm^3^ | Volume of subcompartment representing cortex[^21^](#_ENREF_21) |
| L | 20 | cm | ^f^Length of the spinal fluid (C1) or tissue (C2)[^50^](#_ENREF_50) |
| A | V_1_/L | cm^2^ | Cross-section of spinal CSF space, C1 |
| k1 | 10^-9^ | min^-1^ | ^(This work)^Rate constant for first-order clearance in C1 |
| k2 | 8×10^-6^ | min^-1^ | ^(This work)^Rate constant for first-order clearance in C2 |
| k3 | 10^-9^ | min^-1^ | ^(This work)^Rate constant for first-order clearance in C3 |
| k4 | 1.3×10^-5^ | min^-1^ | ^(This work)^Rate constant for first-order clearance in C4 |
| k5 | 1.5×10^-2^ | min^-1^ | ^(This work)^Rate constant for first-order clearance in C5 |
| k6 | 10^-9^ | min^-1^ | ^(This work)^Rate constant for first-order clearance in C6 |
| A_12_ | $2\sqrt{\pi LV_{2}}$ | cm^2^ | Mass transfer area between C1 and C2 |
| A_13_ | A | cm^2^ | Mass transfer area between C1 and C3 |
| A_15_ | $2\sqrt{\pi LV_{1}}$ | cm^2^ | Mass transfer area between C1 and C5 |
| A_25_ | $2\sqrt{\pi LV_{2}}$ | cm^2^ | Mass transfer area between C2 and C5 |
| A_34_ | 1 | cm^2^ | ^g^Mass transfer area between C3 and C4 |
| A_45_ | 1 | cm^2^ | ^g^Mass transfer area between C4 and C5 |
| A_56_ | 1 | cm^2^ | ^g^Mass transfer area between C5 and C6 |
| U_12_ | 10^-5^ | cm/min | ^(This work)^Mass transfer constant between C1 and C2 |
| U_13_ | 1 | cm/min | ^(This work)^Mass transfer constant between C1 and C3 |
| U_15_ | 8×10^-4^ | cm/min | ^(This work)^Mass transfer constant between C1 and C5 |
| U_25_ | 10^-9^ | cm/min | ^(This work)^Mass transfer constant between C2 and C5 |
| U_34_ | 0.01 | cm/min | ^(This work)^Mass transfer constant between C3 and C4 |
| U_45_ | 10^-5^ | cm/min | ^(This work)^Mass transfer constant between C4 and C5 |
| U_56_ | 0.5 | cm/min | ^(This work)^Mass transfer constant between C5 and C6 |
| β_12_ | 0.004 | N/A | ^(This work)^Stickiness constant between C1 and C2 |
| β_25_ | 10^-9^ | N/A | ^(This work)^Stickiness constant between C2 and C5 |
| β_45_ | 1 | N/A | ^(This work)^Stickiness constant between C4 and C5 |
| β_56_ | 2×10^-3^ | N/A | ^(This work)^Stickiness constant between C5 and C6 |
| φ_pons_ | 0.008 | N/A | ^(This work)^Partition coefficient for pons |
| φ_hipp_ | 0.01 | N/A | ^(This work)^Partition coefficient for hippocampus |
| φ_cerb_ | 0.123 | N/A | ^(This work)^Partition coefficient for cerebellum |
| φ_cort_ | 0.859 | N/A | Partition coefficient for Cortex: φ_cort_ = 1-(φ_pons_+φ_hipp_+φ_cerb_+φ_cort_) |
| *D* | 0.1 | cm^2^/min | ^(This work)^ASO diffusivity in CSF |
| $\dot{F}(\bar{x},t)$ | varying $f\left( t,x \right)$ | ml/min | Infusion flow rate at time t at at location $\bar{x}$ |
| $f\left( t,x \right)$ | varying | ml/min/cm | Local infusion rate obtained by distributing infusate over a small region (2cm) using a truncated, Fourier type, spatial point spread function. |
| V_inj_ | varying | ml | Total injection volume of infusate |

^a^Total CSF volume is assumed to be 11.0 ml[^46^](#_ENREF_46). The spinal part of the CSF is assumed to be 8 ml[^50^](#_ENREF_50).

^b^Spinal tissue volume is approximated based on its reported weight (~6 gm)[^21^](#_ENREF_21).

^c^Cranial CSF volume is estimated by subtracting spinal CSF volume from the total CSF volume.

^d^Assumed to be the sum of the four subcomparments: pons, cerebellum, hippocampus, and cortex.

^e^Represents the central compartment, estimated based on 59 ±15 ml/kg of blood volume[^21^](#_ENREF_21).

^f^Length is estimated based on the lumber, thoracic, and cervical region of the spine[^50^](#_ENREF_50).

^g^A mass transfer area of 1 implies its value was lumped into the mass transfer coefficient.

^(This work)^ Labeled parameters have been identified through calibration as described in the method section (Figs 2-5).
